# Do Vedolizumab trough Levels Predict the Outcome of Subsequent Therapy in Inflammatory Bowel Disease?

**DOI:** 10.3390/biomedicines11061553

**Published:** 2023-05-26

**Authors:** Asaf Levartovsky, Ido Cohen, Chaya Mushka Abitbol, Miri Yavzori, Ella Fudim, Orit Picard, Uri Kopylov, Shomron Ben-Horin, Bella Ungar

**Affiliations:** Department of Gastroenterology, Sheba Medical Center, Tel Hashomer, Affliated to Sackler Medical School, Tel Aviv University, Tel Aviv 69978, Israel

**Keywords:** vedolizumab, trough levels, loss of response, subsequent therapy, therapeutic drug monitoring

## Abstract

**Background**: Vedolizumab trough serum levels have been associated with clinical and endoscopic response in patients with inflammatory bowel disease (IBD). A recent study demonstrated that higher trough levels before dose escalation are associated with favorable outcomes. **Objectives**: We aimed to identify whether vedolizumab trough levels predict outcome of subsequent therapy. **Methods**: This retrospective study included IBD patients consecutively receiving vedolizumab therapy between November 2014 and June 2021. Only patients with a loss of response (LOR) to vedolizumab and available trough drug levels prior to therapy cessation were included. Clinical and endoscopic scores were recorded at 6 and 12 months post switching therapy. **Results**: Overall, 86 IBD patients (51 Crohn’s disease, 35 ulcerative colitis) who discontinued vedolizumab were included; of those, 72 (83.7%) were due to LOR. Upon vedolizumab discontinuation, 66.3% of patients were switched to another biologic therapy. Trough vedolizumab levels at discontinuation due to LOR did not differ between patients with clinical response and LOR regarding subsequent therapy at 6 months [median 33.8 μg/mL (IQR 13.2–51.6) versus 31.7 μg/mL (IQR 9.1–64.8), *p* = 0.9] and at 12 months [median 29.6 μg/mL (IQR 14.3–51.6) versus 34.1 μg/mL (IQR 12.2–64.7), *p* = 0.6]. Patients progressing to subsequent surgery had numerically lower vedolizumab trough levels at LOR compared with patients who were treated with an additional medical therapy (median 14.3, IQR 4–28.2 μg/mL versus 33.5, IQR 13–51.6 μg/mL, *p* = 0.08). **Conclusions**: Vedolizumab trough levels upon LOR do not predict response to subsequent medical therapy; however, lower drug levels may suggest a more aggressive disease pattern and future need for surgery.

## 1. Introduction

Vedolizumab, a humanized gut-specific anti α4β7 integrin monoclonal antibody, is effective in the induction and maintenance of remission in patients with ulcerative colitis (UC) and Crohn’s disease (CD) [1,2,3]. Although long-term studies have shown sustained efficacy over time, loss of response occurs in up to half of CD and UC patients by three years of treatment [4,5].

The role of anti-TNF therapeutic drug monitoring (TDM) in optimizing therapy in patients with IBD is commonly accepted. Higher serum drug trough levels of infliximab and adalimumab have been associated with clinical and endoscopic remission in both UC and CD [6,7,8,9]. As for vedolizumab TDM, studies have been less conclusive. There is evidence of an exposure–efficacy relationship indicating an association between drug levels and clinical, biomarker and mucosal outcomes [10,11,12,13]. However, these reported associations are partial and considerably less comprehensive than the literature regarding TDM of anti-TNF agents. Therefore, vedolizumab trough levels are not routinely measured in clinical practice. Peyrin–Biroulet and colleagues performed a meta-analysis demonstrating that dose intensification (interval shortening) of vedolizumab can achieve regained response in up to 53% of secondary non-responders [14]. Another study showed that lower vedolizumab trough levels may identify individuals likely to respond to dose escalation; however a substantial portion of patients with high levels still responded to interval shortening [15]. Furthermore, a recent multi-center study found that actually higher vedolizumab levels upon loss of response associate with better outcomes of dose intensification [16]. Additionally, it was shown that higher vedolizumab levels were associated with less severe disease, perhaps due to reduced faecal loss and potentially lower drug catabolism. While anti-TNF serum trough levels predict the outcome of subsequent therapy (higher levels were associated with better outcome of out-of-class switch), data as to association of vedolizumab therapy with outcome of subsequent therapy are unavailable [17,18]. Thus, in this study, our aim was to determine whether vedolizumab trough levels can predict outcome of subsequent therapy in the setting of loss of response to vedolizumab. In addition, we aimed to further explore the association between vedolizumab levels and disease severity.

## 2. Materials and Methods

### 2.1. Patient Population

This was a retrospective cohort study of all patients with IBD consecutively receiving vedolizumab therapy between November 2014 and June 2021 at Sheba Medical Center, a tertiary academic center in Israel. Only patients who discontinued vedolizumab therapy and had available drug trough levels prior to therapy cessation (trough levels, right before last vedolizumab infusion) were included.

All sera were prospectively and routinely collected from banked blood of IBD patients immediately before vedolizumab infusions at Sheba medical center.

Exclusion criteria—unavailable vedolizumab trough levels at therapy discontinuation, unavailable clinical data, patients still receiving vedolizumab therapy, age below 18.

For each patient, retrieved data included demographic and clinical data (disease extent, behavior, extraintestinal manifestations, and therapy frequency and dosage, comorbidities), endoscopic reports and laboratory values. CRP levels are considered normal in our medical center if below 5 mg/L.

The primary outcome was the association of vedolizumab trough levels with clinical response to subsequent therapy at 6 and 12 months post switching therapy. Secondary outcomes included the association of vedolizumab trough levels and inflammatory markers.

### 2.2. Measurement of Vedolizumab Concentrations

Integrin α4β7 [2 μg/mL, R&D, Saint Paul, MN, USA] was added to preplated anti-his tag [4 μg/mL, RD, Saint Paul, MN, USA] wells of ELISA plates [Nunc, Roskilde, Denmark]; 100 μ/L of 1:1000 diluted serum was added and incubated for 60 min at room temperature. Plates were then washed and goat anti-human κ chain HRP-labelled antibody [Serotec, Oxford, UK] was added at a concentration of 66 ng/mL for 40 min. The results were read by an ELISA reader EL 800 [Biotek Instruments, Winooski, VT, USA] and expressed as μg/mL. The level of detection of the assay was 3 ng/mL, and the level of quantitation was 3 μg/mL. For assay validation, graded vedolizumab concentrations were added to vedolizumab-negative sera, showing significant correlation of signal optical density with the concentrations added (rho = 1; *p* < 0.0001).

### 2.3. Determination of Anti-Vedolizumab Antibody Concentration

A drug-tolerant ELISA assay using an anti-human λ-chain–conjugated detector antibody was developed. Briefly, 100 μL of 1:500 diluted serum was added to preplated 5 μg/mL vedolizumab (Takeda Pharma, Roskilde, Denmark) and incubated for 60 min. After washing, horseradish–peroxidase-labeled goat antihuman λ-chain antibody (MP Biomedicals, Solon, OH, USA) at a concentration of 33 ng/mL was added for 40 min and reacted with tetramethyl–benzidine substrate. The results were read by ELISA reader EL-800 (Biotek Instruments, Winooski, VT, USA) and expressed as microgram per milliliter equivalents after normalization vs graded concentrations of 9 to 1200 ng/mL of goat anti-human F(ab’)2 fragment antibody (MP Biomedicals, Santa Ana, CA, USA). AVA levels greater than 30 μg/mL equivalent cut-off values were considered positive, as determined by 3 SDs greater than the mean of 30 unexposed controls.

### 2.4. Clinical and Endoscopic Scores

Clinical status was determined by HBI [Harvey-Bradshaw Index] for CD and by SCCAI [Simple Clinical Colitis Activity Index] for UC patients [19,20]. Clinical remission was defined as HBI < 5 for CD patients and SCCAI ≤ 3 for UC patients. Clinical response was defined as a decline of >3 points in HBI score or in SCCAI for CD and UC, respectively. In the minority of patients (5.8%, 5 patients) when scores were unavailable, clinical response was determined by the physician’s global assessment.

Endoscopic assessment was performed using the Mayo endoscopic sub-score for UC. For CD, SES-CD (The Simple Endoscopic Score for Crohn Disease) scores were used. As per scores, all patients’ endoscopies were categorized as: intact endoscopy/mild-moderate disease/severe disease [21].

### 2.5. Statistical Analysis

Continuous variables were expressed as median and interquartile range (IQR) and categorical variables as a percentage. *t*-test and Mann–Whitney testing were used to compare continuous variables and Fisher’s exact test was used for categorical data. All reported *p*-values were two-sided, and a *p*-value less than 0.05 was considered statistically significant. All statistical tests were two-sided, and a *p* value < 0.05 was considered statistically significant. Statistical analysis was performed using the SPSS v23 statistical software (IBM Crop., Armonk, NY, USA). A power analysis was performed to estimate the optimal sample size required in order to detect a statistically significant difference in vedolizumab trough levels between patients responding/not responding to the next intervention/undergoing a surgery or not. For an alpha error defined as 0.05, with a power of 80%, a minimal sample of N = 16 is required for each group to detect a minimal difference of 25% in trough levels.

## 3. Results

**Figure 1:**
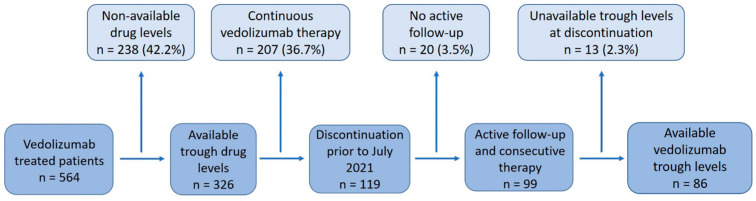
Flow chart showing the study layout.

A total of 564 IBD patients were treated with Vedolizumab at Sheba Medical Center between November 2014 and June 2021. Of those, 119 (21%) discontinued vedolizumab therapy prior to June 2021. A total of 20 patients were excluded due to insufficient follow-up and an additional 13 patients were excluded because of unavailable vedolizumab trough levels at therapy discontinuation (Figure 1). Eventually, 86 patients were included in the cohort, and of those 59.3% (51/86) CD and 40.7% (35/86) UC patients.

Demographics, prior treatment and IBD characteristics are summarized in Table 1. The median age of IBD diagnosis was 26 years (IQR 18–39.5), and a total of 48 patients (55.8%) were males. A total of 53 (61.6%) and 37 (43%) patients were previously treated with infliximab and adalimumab, respectively. Only five (5.8%) patients were priorly treated with ustekinumab. The majority of CD patients (55%) had an ileocolonic disease and 45.3% of patients had an extra-intestinal manifestation, mostly (76%) peripheral arthropathy.

### 3.1. Vedolizumab and Subsequent Therapy

The included patients were treated with vedolizumab for a median of 46.4 weeks (IQR 29.6–83.6) until treatment discontinuation. The majority of patients, 83.7% (72/86), discontinued therapy due to loss of response, and 15.1% (13/86) discontinued due to adverse events. The majority of adverse events were arthralgia (4/13, 30.8%) and skin rashes including erythema nodosum (4, 30.8%) (Table 2).

Median trough vedolizumab levels at discontinuation were 30 µg/mL (IQR 12.5–53). None of the patients had positive anti-vedolizumab antibody levels upon LOR. Sera of patients with trough vedolizumab levels < 10 μg/mL were analyzed for anti-vedolizumab antibodies (*n* = 17). Among these patients, there were no positive anti-vedolizumab antibodies at drug discontinuation (0/17).

At vedolizumab discontinuation, 69.8% of patients underwent dose escalation receiving therapy every 4 weeks. A numerical difference was seen for higher vedolizumab trough levels upon LOR among patients who underwent interval shortening compared to patients who received vedolizumab every 8 weeks at LOR [median 33.8 μg/mL (IQR 13.6–60.4) versus 14.6 μg/mL (IQR 7.5–34.4), respectively, *p* = 0.06].

### 3.2. Outcome of Subsequent Medical (Non-Surgical) Therapy

**Figure 2:**
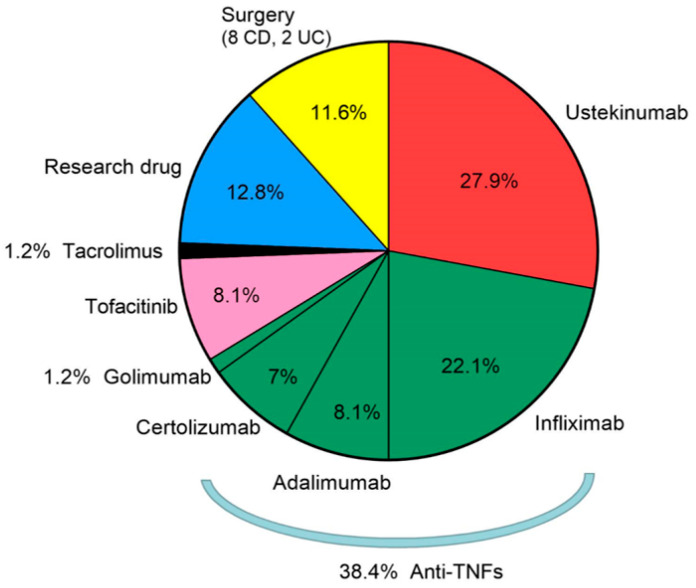
Subsequent therapeutic strategies after vedolizumab discontinuation among the study cohort. TNF—Tumor necrosis factor, CD—Crohn’s disease, UC—ulcerative colitis.

**Figure 3:**
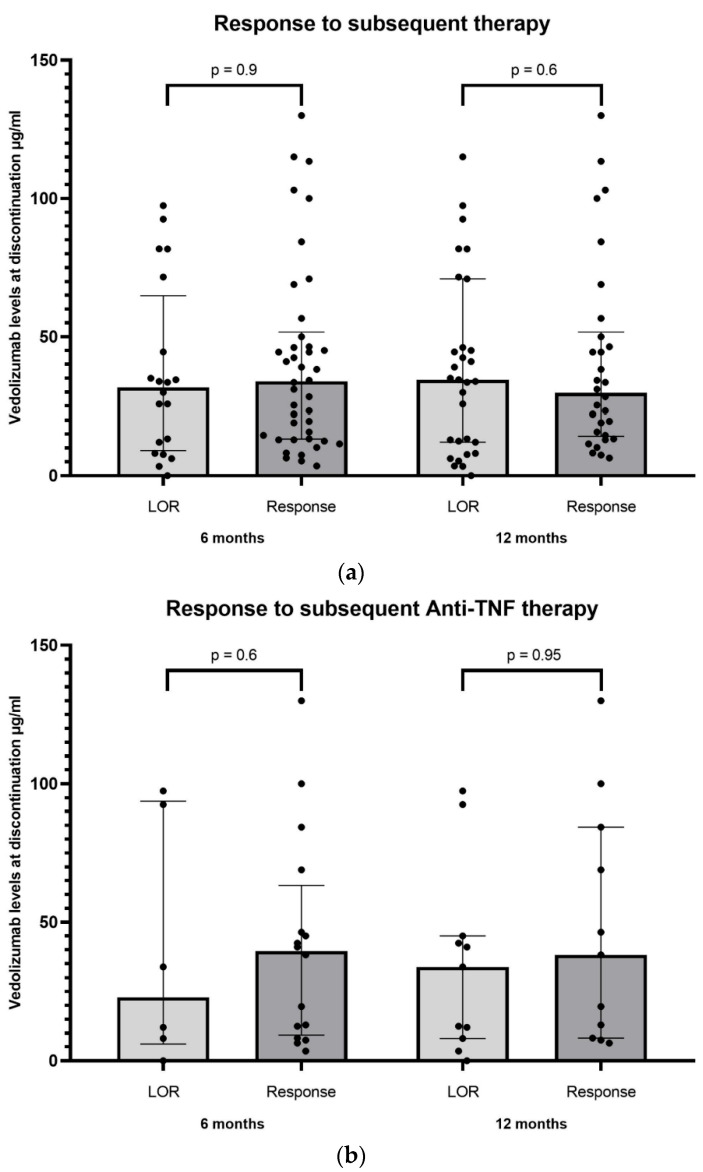
Vedolizumab discontinuation trough levels in association with response to subsequent all non-surgical therapy (**a**) and anti-TNF therapy (**b**) at 6 and 12 months post discontinuation. Data presented as median and IQR. TNF—Tumor necrosis factor.

The subsequent therapeutic strategies after vedolizumab discontinuation among the study cohort are detailed in Figure 2. Among patients with loss of response (LOR) to vedolizumab, there was no significant difference in vedolizumab levels upon discontinuation between patients with clinical response (42/62) and LOR (20/62) to subsequent medical therapy after 6 months [median 33.8 μg/mL (IQR 13.2–51.6) versus 31.7 μg/mL (IQR 9.1–64.8), *p* = 0.9]. Similar findings were observed for patients responding (30/62) and losing response (32/62) to subsequent medical therapy after 12 months [median 29.6 μg/mL (IQR 14.3–51.6) versus 34.1 μg/mL (IQR 12.2–64.7), *p* = 0.6]. The same analysis was performed in the subset of patients who were treated with subsequent anti-TNF therapy (Figure 3a,b). In a subsequent Ustekinumab treatment analysis (24/86 patients), no significant differences were seen in vedolizumab levels upon discontinuation between patients with clinical response and LOR after 6 and 12 months of ustekinumab therapy (*p* = 1, 0.68, respectively).

A sub-analysis was performed for patients who underwent vedolizumab dose interval shortening prior to discontinuation, receiving vedolizumab every 4 weeks. This analysis did not yield any significant differences in response to subsequent therapy between the groups at 6 and 12 months (*p* = 0.6, 0.94, respectively).

When analyzing the results only for CD patients (37/62), there were no differences in vedolizumab levels upon discontinuation between patients with clinical response and LOR at 6 months [median 34.2 μg/mL (IQR 13.8–46.2) versus 21.6 μg/mL (IQR 6.6–34.8), *p* = 0.8] and 12 months [median 33.8 μg/mL (IQR 16.6–54.1) versus 29.9 μg/mL (IQR 6.9–43.4), *p* = 0.85] of the subsequent therapy. For UC patients (25/62), as in line with the above results, no significance was demonstrated in vedolizumab levels between responders and non-responders (Supplementary Table S1). The results of these sub-analyses could be attributed to their limited sample sizes.

Endoscopic scores, when available, were assessed after 6 months of subsequent medical therapy (28/62); of them, 13 were UC and 15 were (3 colonic and 8 ileo-colonic) CD patients. A total of 6 patients (21.4%) had a normal colonoscopy, 12 patients (42.9%) had a mild-moderate disease and 10 patients (35.7%) had severe disease. No significant differences were observed in vedolizumab levels upon discontinuation between normal (6, 21.4%), mild-moderate (12, 42.9%) and severe (10, 35.7%) endoscopic scores [normal-median 47.8 μg/mL (IQR 27.3–93.8), mild-moderate-median 20.6 μg/mL (IQR 10.9–32.9), severe-median 29.4 μg/mL (IQR 10.6–54.5), *p* = 0.13].

**Figure 4:**
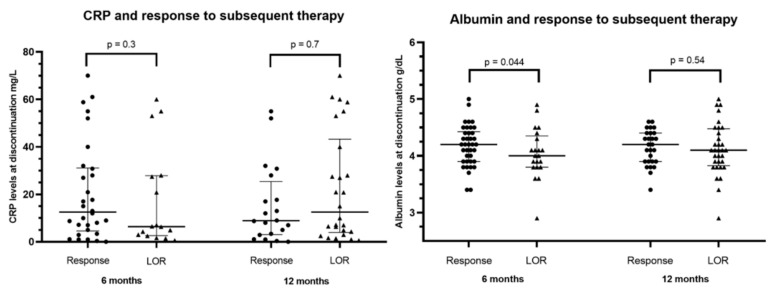
Markers of inflammation (CRP and albumin) assessed at time of loss of response to vedolizumab and response to subsequent non-surgical therapy after 6 and 12 months. Data presented as median and IQR. CRP—C-reactive protein.

Apart from drug levels, additional markers of inflammation were assessed at time of loss of response to vedolizumab. Albumin levels were significantly lower among patients with loss of response compared to patients responding to subsequent therapy at 6 months (median 4, IQR 3.8–4.25 g/dL versus 4.2, IQR 3.9–4.5 g/dL, *p* = 0.044). No difference in albumin levels was observed after 12 months (Figure 4). No significant differences were observed in CRP values among patients responding and losing response to subsequent medical therapy at 6 months and at 12 months.

When analyzing the results separately for CD and UC patients, no significant differences were observed in CRP and albumin values among patients responding and losing response to subsequent medical therapy at 6 months and at 12 months (Supplementary Table S1).

### 3.3. Subsequent Surgical Therapy

An additional analysis compared patients with LOR to vedolizumab treated with subsequent medical therapy (62/72, 86.1%) as opposed to surgical therapy. A total of 10/72 (13.9%) patients underwent abdominal surgery as their next therapeutic intervention after stopping vedolizumab. Of these, eight CD patients (six ileocolectomies, two small bowel resection) and two UC patients (one subtotal colectomy, one Ileal pouch-anal anastomosis). This analysis demonstrated a trend towards lower vedolizumab trough levels at discontinuation among patients undergoing subsequent surgery compared with patients referred for medical therapy (median 14.3, IQR 4–28.2 μg/mL versus 33.5, IQR 13–51.6 μg/mL, respectively, *p* = 0.08). Analyzing only the CD patients, vedolizumab trough levels at discontinuation were non-significantly lower compared with patients referred for medical therapy (median 13.2, IQR 2.4–29.1 μg/mL versus 33.5, IQR 12.7–44.7 μg/mL, respectively, *p* = 0.27).

In the interval shortening sub-analysis, this comparison was significant as patients undergoing subsequent surgery had lower vedolizumab trough levels (median 14.7, IQR 8.6–30.1 μg/mL versus 41, IQR 19.1–71.6 μg/mL, *p* = 0.03).

**Figure 5:**
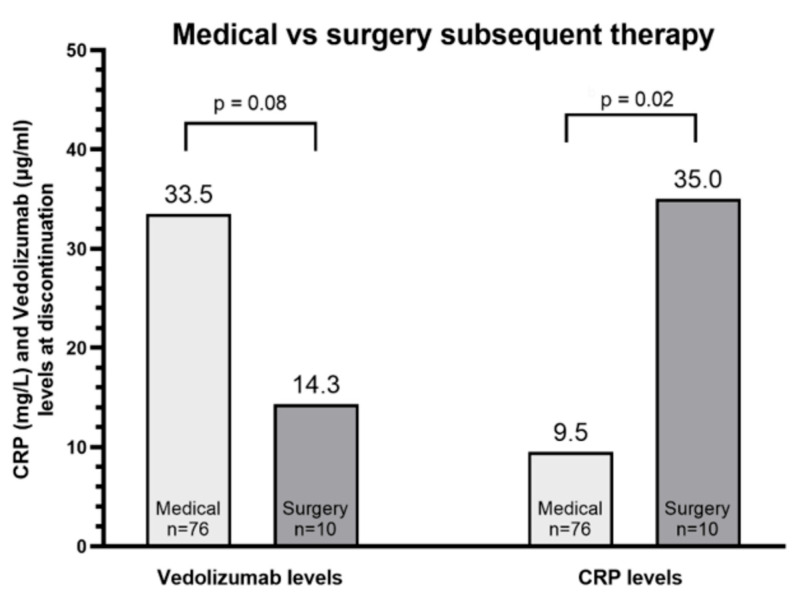
Vedolizumab and CRP levels at discontinuation of therapy among patients treated with subsequent non-surgical and surgical therapy. CRP—C reactive protein, vs—versus.

CRP levels at vedolizumab cessation were significantly higher among patients undergoing subsequent surgery [median 35 mg/L, IQR 18.9–44.1 vs 9.5 mg/L, IQR 3.3–28.7, *p*= 0.02]. These results are depicted in Figure 5. A numerical trend was seen for higher albumin levels at vedolizumab LOR among patients undergoing subsequent medical (median 4.1, IQR 3.9–4.4 g/dl) compared to subsequent surgical therapy (median 3.9, IQR 3.17–4.1 g/dl, *p* = 0.24). These results were similar when analyzing solely the CD patients.

## 4. Discussion

This cohort study of patients with IBD on maintenance vedolizumab treatment aimed to determine whether trough levels upon LOR can predict outcome of subsequent therapy. Our findings demonstrate that vedolizumab TDM has no role in predicting clinical response to subsequent medical therapy; however, it may serve as an indirect marker of disease severity and prognosis. Lower drug levels may suggest a more aggressive disease pattern and future need for surgery. Data emerging in recent years support an association between vedolizumab drug levels and long-term outcomes. Data from the GEMINI trials revealed the fundamental relationship between higher vedolizumab concentration at week 6 and increased rates of clinical response and remission [1,2,3]. Osterman et al. and Vande Casteele et al. showed that higher vedolizumab concentrations at an early stage as week 6 can predict clinical remission at weeks 14 and 52 [22,23]. Guidi et al. predicted treatment persistence over the first year of vedolizumab treatment, based on high trough levels at week 14 [24]. Vedolizumab drug levels have been associated with endoscopic outcomes as well [12,25,26,27].

Vedolizumab dose optimization has become a commonly used strategy to regain therapeutic effect upon insufficient response or LOR. Loftus et al. showed a higher response and remission rates following intervals shortened to every 4 weeks [5]. Additional studies including a meta-analysis showed that upon LOR, dose intensification to every 4 weeks resulted in regained clinical response with higher trough levels in half of the patients [14,28]. Conflicting findings were published as well. Vermeire et al. demonstrated stable clinical remission rates despite a significant decrease in trough levels following de-escalation from vedolizumab every 4 weeks to every 8 weeks [29]. Vaughn et al. showed that although lower vedolizumab levels may predict response to dose escalation, patients with high levels may respond as well [15]. Based on data from the ENTERPRET study, non-responders at week 6 who received dose-optimized vedolizumab at week 10 had similar rates of mucosal healing and clinical remission at week 30 when compared with patients receiving standard regimen [30]. In line with these studies, our group showed similar or higher pre-optimization vedolizumab levels in patients with LOR who responded to dose optimization compared with non-responding patients [16]. Thus, despite pre-supposed utility of dose optimization, it seems that LOR to vedolizumab does not stem from a pharmacokinetic mechanism of drug failure. On these grounds, we explored whether the presence of high trough levels upon LOR to vedolizumab could predict outcome to subsequent therapy. Our findings demonstrated similar drug levels upon discontinuation of vedolizumab between patients with long-term clinical response and those with LOR after initiation of various biological agents. Anti-vedolizumab antibodies were not detected in this study and were not associated with therapy outcome. These findings in addition to the aforementioned studies argue against an association between vedolizumab drug levels and the probability of future response. Thus, although vedolizumab levels taken at an early stage of therapy may be associated with future outcomes, they currently have no apparent role in predicting response to future subsequent therapy. Notably, the majority of the cohort were non-responders to prior anti-TNF therapy (66/86, 76.7%), and subsequent therapy upon LOR to vedolizumab consisted mostly of additional agents targeting different mechanisms (ustekinumab, tofacitinib, research drug and surgical management). However, a sensitivity analysis restricted only to patients who were swapped to anti-TNFs showed similar results.

An additional aim was to further explore the relationship between vedolizumab levels and disease severity. Higher albumin levels were previously associated with increased vedolizumab trough concentrations and a higher probability of remission [3,10,31]. Schultze et al. demonstrated that CRP levels declined following vedolizumab initiation among patients with CD and that lower levels were associated with higher drug trough levels [32]. Dulai and colleagues devised and validated a clinical decision tool for identifying predictors of high probability for response to vedolizumab [33]. In our study, we examined whether CRP and albumin may predict response to subsequent therapy. While there was no significant difference in CRP levels between the groups, albumin levels were lower among patients eventually not responding to subsequent therapy. These findings are compatible with the above-mentioned studies and imply that patients with low albumin levels at LOR have a more severe and difficult-to-treat disease. Interestingly, this study also gives an insight into the gap between medical management and surgery. A trend for lower vedolizumab levels was demonstrated among patients undergoing subsequent surgery. Furthermore, vedolizumab levels were significantly lower among patients undergoing vedolizumab interval shortening. This signifies that perhaps patients referred to surgery rather than an additional therapeutic agent have a more severe disease pattern associated with lower vedolizumab trough levels. Indeed, CRP levels at vedolizumab LOR were significantly higher among patients undergoing subsequent surgery, reflecting a more severe disease activity status. Studies regarding vedolizumab TDM and postoperative response are scarce, showing that in contrast to anti-TNF, preoperative vedolizumab levels have no major effect on postoperative morbidity and outcomes in IBD [34]. Although the evidence does not support vedolizumab levels as a predictor of post-surgical outcomes, perhaps there is a role for predicting further progression to surgical therapy after LOR to biologic therapy. This further strengthens the notion that vedolizumab levels may serve as an indirect marker of severity of inflammation.

Several limitations should be acknowledged including the retrospective nature of the data collection from more than 560 patients, who were treated with vedolizumab within the designated timeframe. Unknown co-variants cannot be ruled out. Second, although the study includes 86 patients, in losing response to vedolizumab with available trough levels upon LOR, they were further subjected to various pharmacological therapies, limiting sample size and possibly having an impact on the absence of a clear signal between vedolizumab trough levels and response to subsequent therapy. Furthermore, the relatively low rate of patients progressing to surgical therapy could limit the power to detect differences therefore; the results, of the study should be taken with caution. In any case, further large-scale studies are required.

In conclusion, this study demonstrated that vedolizumab trough levels upon LOR do not predict clinical response to subsequent medical therapy. As opposed to anti-TNF treatment algorithm, higher drug levels were not associated with better outcomes of therapy switching. Furthermore, lower drug levels may suggest a more aggressive disease pattern and future need for surgery, as per CRP and albumin levels. This study further emphasizes that vedolizumab levels may serve as an indirect marker of disease severity and the degree of inflammation and that higher vedolizumab levels may indicate a less severe disease pattern.

## Figures and Tables

**Table 1:** Study cohort demographics and IBD characteristics.

**Study Population** ***n* = 86**	
Age of diagnosis, *n* (median, IQR)	26 (18, 39.5)
Male, *n* (%) Female, *n* (%)	48 (55.8) 38 (44.2)
CD, *n* (%) UC, *n* (%)	51 (59.3) 35 (40.7)
CD extent, *n* (% of CD patients) L1 (ileal) L2 (colonic) L3 (ileo-colonic) Perianal disease	19 (37.2) 4 (7.8) 28 (55) 16 (31.4)
CD behavior, *n* (% of CD patients) B1 (non-stricturing and non-penetrating) B2 (stricturing) B3 (penetrating) B2 + B3	13 (25.5) 16 (31.4) 18 (35.3) 4 (7.8)
UC extent, *n* (% of UC patients) E1 (proctitis) E2 (left sided colitis) E3 (right sided colitis)	0 (0) 13 (37.1) 22 (62.9)
Extra-intestinal manifestations, *n* (%)	39 (45.3)
Smoking (current or past), *n* (%)	19 (22.1)
**Past IBD Treatment**	
Immunomodulator, *n* (%)	56 (65.1)
5-ASA derivatives, *n* (%)	49 (57)
Infliximab and reason for cessation, *n* (%) LOR Immunogenicity Adverse event Compliance Other	53 (61.6) 26 (49.1) 13 (24.5) 11 (20.7) 0 (0) 3 (5.7)
Adalimumab and reason for cessation, *n* (%) LOR Immunogenicity Adverse event Compliance Other	37 (43) 23 (62.1) 6 (16.2) 5 (13.5) 1 (2.7) 2 (5.4)
Ustekinumab and reason for cessation, *n* (%) LOR	5 (5.8) 5 (100)

ASA—Amino-salicylic acid, CD—Crohn’s disease, IBD—inflammatory bowel disease, IQR—interquartile range, LOR—loss of response, UC—Ulcerative colitis.

**Table 2:** Vedolizumab treatment and subsequent therapy.

**Vedolizumab Treatment**	
Duration of treatment, median weeks, *n* (IQR)	46.4 (29.6, 83.6)
Reason for therapy cessation, *n* (%) LOR Adverse event Compliance	72 (83.7) 13 (15.1) 1 (1.2)
Adverse events leading to therapy cessation, *n* (% of adverse events) Erythema Nodosum Arthralgia Paraesthesia Rash Acute otitis media Headache Recurrent pharyngitis Elevated liver enzymes	1 (7.7) 4 (30.7) 1 (7.7) 3 (23.1) 1 (7.7) 1 (7.7) 1 (7.7) 1 (7.7)
Additional therapy on stop, *n* (%) Steroids Immunomodulators 5-ASA derivatives	28 (32.5) 9 (10.4) 24 (27.9)
Last drug levels, μg/mL (IQR)	30 (12.5, 53)
Vedolizumab dose interval on discontinuation, *n* (%) 4 weeks 8 weeks	60 (69.8) 26 (30.2)
CRP on stop, mg/L (IQR)	11 (4.6, 34.25)
**Sequential Treatment, *n* (%)**	
Infliximab Adalimumab Ustekinumab Certolizumab Golimumab Tofacitinib Tacrolimus Research Surgery	19 (22.1) 7 (8.1) 24 (27.9) 6 (7) 1 (1.2) 7 (8.1) 1 (1.2) 11 (12.8) 10 (11.6)
Duration between vedolizumab cessation and initiation of subsequent therapy, median weeks, *n* (IQR)	8.8 (5.32, 21.1)
Reason for cessation of subsequent therapy, *n* (%) LOR Immunogenicity Adverse event Compliance Other Therapy ongoing	39 (51.3) 2 (2.6) 9 (11.8) 1 (1.3) 2 (2.6) 24 (31.6)

ASA—Amino-salicylic acid, CRP—C-reactive protein, IQR—interquartile range, LOR—loss of response.

## Data Availability

Data is unavailable to open access due to privacy or ethical restriction. To obtain any data of interest, please contact corresponding author.

## Supplementary Materials

The following supporting information can be downloaded at: https://www.mdpi.com/article/10.3390/biomedicines11061553/s1, Table S1: Vedolizumab trough levels and biomarker levels of CD and UC patients at LOR of sequential therapy.
