# Supplementary material for: Do Vedolizumab trough Levels Predict the Outcome of Subsequent Therapy in Inflammatory Bowel Disease?
Source: Biomedicines. 2023 May 26;11(6):1553. doi: 10.3390/biomedicines11061553 (PMC10295644; doi:10.3390/biomedicines11061553)
Supplement: Supplementary file 1 [file biomedicines-11-01553-s001.zip › biomedicines-2346594-supplementary.pdf]

Supplementary Materials

Table S1. Vedolizumab trough levels and biomarker levels of CD and UC patients at LOR of sequential therapy.

CD patients	51/86 patients
**Vedolizumab trough levels upon discontinuation**	
6 months of subsequent therapy (n=37)	
LOR (n=12)	21.6 (6.6-34.8)
Responders (n=25)	34.2 (13.8-46.2)
P value	0.8
12 months of subsequent therapy (n=37)	
LOR (n=21)	29.9 (6.9-43.4)
Responders (n=16)	33.8 (16.6-54.1)
P value	0.85
Subsequent medical therapy (n=37)	33.5 (12.7-44.7)
Subsequent surgical therapy (n=8)	13.2 (2.4-29.1)
P value	0.27
**CRP levels upon discontinuation**	
6 months of subsequent therapy (n=25)	
LOR (n=8)	27.7 (9.9-54.5)
Responders (n=17)	27 (8.5-46)
P value	1
12 months of subsequent therapy (n=25)	
LOR (n=16)	27.7 (11.2-57.8)
Responders (n=9)	17 (6-31.4)
P value	1
Subsequent medical therapy (n=25)	27.4 (8.5-52.5)
Subsequent surgical therapy (n=8)	30.5 (18.1-45.5)
P value	0.7
**Albumin levels upon discontinuation**	
6 months of subsequent therapy (n=34)	
LOR (n=12)	4 (3.6-4.4)
Responders (n=22)	4.2 (4.0-4.5)
P value	0.4
12 months of subsequent therapy (n=34)	
LOR (n=21)	4.1 (3.8-4.5)
Responders (n=13)	4.3 (4-4.4)
P value	0.26
Subsequent medical therapy (n=34)	4.2 (3.8-4.5)
Subsequent surgical therapy (n=8)	3.9 (3.3-4.3)
P value	0.3
**UC patients**	**35/86 patients**
**Vedolizumab trough levels upon discontinuation**	
6 months of subsequent therapy (n=25)	
LOR (n=8)	52.5 (25.7-89.8)
Responders (n=17)	28.3 (11.7-69.9)
P value	1
12 months of subsequent therapy (n=25)	

LOR (n=11)	70.9 (25.7-92.5)
Responders (n=14)	22.7 (9.7-54.7)
P value	0.2
**CRP levels upon discontinuation**	
6 months of subsequent therapy (n=21)	
LOR (n=8)	27.7 (9.9-54.5)
Responders (n=13)	27 (8.5-46)
P value	1
12 months of subsequent therapy (n=21)	
LOR (n=10)	27.7 (11.2-57.8)
Responders (n=11)	17 (6-31.4)
P value	1
**Albumin levels upon discontinuation**	
6 months of subsequent therapy (n=25)	
LOR (n=8)	4 (3.6-4.4)
Responders (n=17)	4.2 (4.0-4.5)
P value	0.4
12 months of subsequent therapy (n=25)	
LOR (n=11)	4.1 (3.8-4.5)
Responders (n=14)	4.3 (4-4.4)
P value	0.26
